# A Thermodynamic Study on Information Power in Communication Systems

**DOI:** 10.3390/e26080650

**Published:** 2024-07-30

**Authors:** Litao Yan, Xiaohu Ge

**Affiliations:** School of Electronic Information and Communications, Huazhong University of Science and Technology, International Joint Research Center of Green Communications and Networking, Wuhan 430074, China; litaoyan@hust.edu.cn

**Keywords:** heat engine, entropy production, information transmission, energy transmission, MIMO

## Abstract

Modern information theory pioneered by Shannon provides the mathematical foundation of information transmission and compression. However, the physical (and especially the energetic) nature of the information has been elusive. While the processing of information incurs inevitable energy dissipation, it is possible for communication systems to harness information to perform useful work. In this article, we prove that the thermodynamic cost (that is, the entropy production of the communication system) is at least equal to the information transmitted. Based on this result, a model of a communication heat engine is proposed, which can extract work from the heat bath by utilizing the transmission of information. The communication heat engine integrates the manipulation of both energy and information so that both information and power may be transmitted in parallel. The information transmission rate and the information power of the communication heat engine are derived from a pure thermodynamics argument. We find that the information power of the communication heat engine can be increased by increasing the number of communication channels, but the absolute energy efficiency of the heat engine first increases and then decreases after the number of channels of the system exceeds a threshold. The proposed model and definitions provide a new way to think of a classical communication system from a thermodynamic perspective.

## 1. Introduction

The field of communication engineering has witnessed significant advancements and transformations since Claude Shannon’s groundbreaking work in the mid-20th century [1]. Shannon’s work provided a solid mathematical foundation for the information theory, based on which sophisticated coding schemes [2,3,4] and advanced communication technologies [5,6,7,8,9,10] are developed to greatly improve the reliability and efficiency of information transmission systems. These improvements have significantly advanced information transmission and processing, contributing to the era of increased connectivity and the widespread adoption of wireless communications [11]. However, with the rapid increase in data traffic and the proliferation of mobile devices, the energy consumption of communication systems has emerged as a critical concern for designing future communication systems. When optimizing the energy efficiency of communication systems, traditional studies generally handle energy and information separately. By treating energy and information as independent entities, mathematical optimizations have difficulty in capturing the potential synergies that could be achieved through a more holistic approach. There are a few studies which explore the relationships between information and the energy of communication processes from a more fundamental perspective [12,13]. The energy dissipation of a mobile communication system has been analyzed using tools from stochastic thermodynamics [14], and it has been shown that the minimization of entropy production in communications systems (and hence, their energy efficiency) can be addressed systematically [15]. However, the thermodynamic role of information in a communication system remains elusive for researchers in the field of communication.

The physical nature of information and communication has been studied in the field of statistical physics for many years. By considering thermodynamic entropy and information entropy as the same concept, E. T. Jaynes proposed the maximum entropy principle and established the framework of statistical physics from the perspective of information theory [16,17,18]. It has been shown that information theory provides a foundation for understanding thermodynamic concepts, enabling a direct and simple derivation of classical and statistical thermodynamics [19]. Using tools from information theory, the relationship between entropy change, free energy, and coding conversion costs in the Carnot cycle was analyzed, demonstrating how information theory can help understand details and possible asymmetries in thermodynamic cycles [20]. On the other hand, the Gedanken experiment of Maxwell’s demon raised an extensively argued topic concerning the information-to-energy conversion [21], which further led to the landmark philosophy of Landauer’s principle [22]. Landauer’s principle demonstrates that there is inevitable energy dissipation when information is erased or processed in a logical irreversible way, providing significant insights into the study of the relationship between information and energy [23]. Assuming both logical and thermodynamic reversibilities [24] of bit operations, Landauer stated that the energy cost of transmitting each bit of information can theoretically be reduced to zero [25,26]. Nevertheless, the energy dissipation of real-world communication processes needs to be analyzed in a more formal and general way, where the dynamic movement of information must be considered [27]. Recent breakthroughs in nonequilibrium thermodynamics (the fluctuation theorems [28] and the Jarzynski equality [29]) and the development of stochastic thermodynamics [30] have made the quantitative analysis of the thermodynamics of information transmission and processing possible. The Jarzynski equality was generalized to a model of information ratchet, where positive work could be extracted through measurement and feedback control [31]. Quantifying information processing through differences in free energy, a thermodynamic model of bounded rational decision-making was proposed, addressing the trade-off between expected utility and information-processing costs [32]. Furthermore, the conversion of information to work has been experimentally verified in small thermodynamic systems [33] and quantum systems [34,35].

Despite the various connections between information theory and thermodynamics, the practical application of physical principles to redefine future paradigms of communication systems has been less prevalent. Here, motivated by the recent development of the thermodynamics of information, we derive the lower bound of the energy dissipation of a communication system within the framework of nonequilibrium thermodynamics. The energy dissipation compensates the entropy decrease of the heat source, leading us to think of classical communication systems from a thermodynamic and energetic perspective, i.e., the communication heat engine. By treating information and energy from a unified perspective, the proposed model of a communication heat engine introduces an approach for energy utilization through the mechanism of communication. Therefore, a new insight of the parallelizable transmission of information and power in the communication system is provided, and the energy efficiency of the communication system is redefined. We derived the information transmission rate and the maximal power of the communication heat engine. Our quantitative results show that by adopting a multi-channel communication scheme, the absolute energy efficiency of the communication heat engine first increases then decreases after the number of channels exceeds a threshold in communication systems. Moreover, the impact of inter-channel interference on the performance of the communication heat engine is analyzed and simulated.

## 2. Minimum Energy Dissipation of Communication System

In communication systems, the signal and transmission medium both involve energy transformations and state change characteristics of nonequilibrium systems. Hence, a realistic communication system operates under nonequilibrium conditions and generates unavoidable energy dissipation. The magnitude of energy dissipation depends on the specific realization and operating state of the system. In order to achieve the minimum energy dissipation of the communication system, here, we consider a simplified communication system consisting of a transmitter, a communication channel, and a receiver. The information sent by the transmitter is received by the receiver through the communication channel, and the system is assumed to be in a room-temperature *T* environment. The channel is assumed to introduce the thermal noise with the power spectral density kT to the transmitted signal, where *k* is the Boltzmann constant. We use X and Y to represent the physical state space of the transmitter and receiver, respectively, and the physical states of the transmitter and receiver are represented by the random variables *X* and *Y*, respectively. When the transmitter is configured in a specific state x∈X, the receiver will evolve to a corresponding state y∈Y under coupling to the communication channel. The receiver can also be described by an energy function, i.e., the Hamiltonian E(y|x), which is the energy of the receiver in state *y* given the transmitter in state *x*. Due to the channel noise, the equilibrium states of transmitter and receiver may not be the same. When the receiver is under an equilibrium condition, the probability distribution of the receiver state given the state of the transmitter follows the Boltzmann distribution [36], i.e., $p\left(y|x\right)=e^{-\beta E(y|x)}/Z$, where β=1/kT, and the term $Z=\sum_{y}e^{-\beta E(y|x)}$ is the partition function for a particular transmitter state *x*. For convenience of calculation, we set k=1. At time t=0, the communication system is in equilibrium, and the state of the transmitter $X_{0}$ and the state of the receiver $Y_{0}$ are assumed to be $x_{0}$ and $y_{0}$, respectively. At time t=1−Δt, the state of the transmitter changes from $x_{0}$ to $x_{1}$, the potential provided by the transmission signal changes instantaneously, and the Hamiltonian of the receiver can be denoted as $E(y_{0}|x_{1})$. Then, within the time duration Δt, the receiver evolves to a new equilibrium state under the potential of the transmission signal and the heat bath, reaching the Boltzmann distribution at time t=1, while the state of the transmitter within Δt is assumed to be kept unchanged. At time t=1, the specific state of the receiver is denoted by $y_{1}$, and the Hamiltonian of the receiver given the state of the transmitter is denoted by $E(y_{1}|x_{1})$. The process of the receiver from the state at time t=1−Δt to the equilibrium state is an irreversible thermodynamic process. If we denote the heat dissipated by the receiver into the environment during the nonequilibrium process as *q*, the heat dissipation can be calculated as $q=E\left(y_{0}|x_{1}\right)-E\left(y_{1}|x_{1}\right)$ based on the law of energy conservation. For all possibilities of the states, the average heat dissipation of the receiver can be expressed as $Q=\sum_{x_{1},y_{0},y_{1}}p(x_{1},y_{0},y_{1})q$, where $p(x_{1},y_{0},y_{1})$ is the joint probability of the state of the transmitter at time t=1, and the state of the receiver at t=0 and t=1. It should be pointed out that the time t=1 does not necessarily mean t=1 second. In this article, t=1 only represents the time when the use of the channel ends, i.e., the state of the communication system reaches equilibrium, and the interval between two successive state evolutions of the system is equivalent to the time when the channel is used.

To carry on the thermodynamic analysis, another thermodynamic quantity we need to know is the nonequilibrium thermodynamic entropy of the receiver given the state of the transmitter. The nonequilibrium thermodynamic entropy is defined by the Shannon entropy multiplied by the Boltzmann constant *k*, where the Shannon entropy is measured in nats [37]. Therefore, the thermodynamic entropy of the receiver given the state of the transmitter is $S\left(Y|X\right)=kH\left(Y|X\right)=-\sum_{x,y}p(x,y)\log p(y|x)$, where H(Y|X) is the conditional Shannon entropy of *Y* given *X*, and the logarithm is the base-e logarithm. The average heat dissipation *Q* and the entropy change in the receiver ΔS satisfy the entropy balance equation

(1) $$\Delta S=S_{G}-\frac{Q}{T},$$

where we define the entropy change as $\Delta S=S\left(Y_{1}|X_{1}\right)-S\left(Y_{0}|X_{0}\right)$, with $S(Y_{0}|X_{0})$ and $S(Y_{1}|X_{1})$ referring to the conditional thermodynamic entropy of the receiver given the transmitter state at the times t=0 and t=1, respectively. $S_{G}$ is the entropy production, which is a quantity that measures how irreversible a thermodynamic process is. It should be noted that the transmitter is assumed to be directly controlled by an agent to send the information to the receiver. Therefore, the entropy production is related to the entropy change in the receiver given the transmitter state, and the entropy change in the entropy balance equation is averaged over all the ways that the transmitter is controlled.

**Theorem** **1.** *In a communication process, the entropy production of the receiver is not less than the mutual information between the transmitter and the receiver.*

**Proof.**  We can denote the state of the receiver and the state of the transmitter at time t=1 as random variables $Y_{1}$ and $X_{1}$, respectively. The entropy change in the receiver between times t=0 and t=1 is

(2) $$\begin{array}{cc} \Delta S & =S\left(Y_{1}|X_{1}\right)-S\left(Y_{0}|X_{0}\right)\\ \; & =-\sum_{x_{1}}\sum_{y_{1}}p\left(x_{1},y_{1}\right)\log p\left(y_{1}|x_{1}\right)+\sum_{x_{0}}\sum_{y_{0}}p\left(x_{0},y_{0}\right)\log p\left(y_{0}|x_{0}\right)\\ \; & =\sum_{x_{0}}\sum_{y_{0}}\sum_{x_{1}}\sum_{y_{1}}p\left(x_{0},y_{0}\right)p\left(x_{1},y_{1}\right)\log\frac{p(y_{0}|x_{0})}{p(y_{1}|x_{1})}. \end{array}$$

Assume that the probabilities of the receiver states are independent. Hence, the joint probability $p\left(y_{0},x_{1},y_{1}\right)=p\left(y_{0}\right)p\left(x_{1},y_{1}\right)$ and the average heat dissipation of the receiver can be expressed as $Q=\sum_{x_{0},y_{0},x_{1},y_{1}}p\left(x_{0},y_{0}\right)p\left(x_{1},y_{1}\right)q$. Based on the entropy balance equation, the entropy production of the receiver is

(3) $$\begin{array}{cc} S_{G} & =\Delta S+\frac{Q}{T}\\ \; & =\sum_{x_{0}}\sum_{y_{0}}\sum_{x_{1}}\sum_{y_{1}}p\left(x_{0},y_{0}\right)p\left(x_{1},y_{1}\right)\log\frac{p(y_{0}|x_{0})}{p(y_{0}|x_{1})}\\ \; & =\sum_{x_{0}}\sum_{y_{0}}p\left(x_{0},y_{0}\right)\log p\left(y_{0}|x_{0}\right)-\sum_{x_{0}}\sum_{y_{0}}p\left(x_{0},y_{0}\right)\sum_{x_{1}}p\left(x_{1}\right)\log p\left(y_{0}|x_{1}\right). \end{array}$$

Because the function f(x)=−logx is convex, upon applying Jensen’s inequality, the last term of Equation (3) satisfies

(4) $$\begin{array}{cc} -\sum_{x_{0}}\sum_{y_{0}}p\left(x_{0},y_{0}\right)\sum_{x_{1}}p\left(x_{1}\right)\log p\left(y_{0}|x_{1}\right) & \geq -\sum_{x_{0}}\sum_{y_{0}}p\left(x_{0},y_{0}\right)\log\sum_{x_{1}}p\left(x_{1}\right)p\left(y_{0}|x_{1}\right)\\ \; & =-\sum_{x_{0}}\sum_{y_{0}}p\left(x_{0},y_{0}\right)\log p\left(y_{0}\right). \end{array}$$

Substituting Equation (4) into Equation (3), we obtain

(5) $$\begin{array}{cc} S_{G} & \geq \sum_{x_{0}}\sum_{y_{0}}p\left(x_{0},y_{0}\right)\log\frac{p(y_{0}|x_{0})}{p(y_{0})}\\ \; & =\sum_{x_{0}}\sum_{y_{0}}p\left(x_{0},y_{0}\right)\log\frac{p(x_{0},y_{0})}{p\left(x_{0}\right)p\left(y_{0}\right)}\\ \; & =I(X;Y), \end{array}$$

which shows that the entropy production of the receiver is no less than the mutual information between the transmitter and receiver. □

Due to the non-negative nature of the mutual information, $S_{G}\geq I\left(X;Y\right)\geq 0$ is obtained. This result also accords with the second law of thermodynamics, which says that the entropy production in the thermodynamic process is non-negative. For a practical communication system, there is non-zero information transmission between the transmitter and the receiver, i.e., I(X;Y)>0. Hence, the total entropy production of the communication system is always greater than zero, meaning that the system is working under the thermodynamic nonequilibrium condition. From another perspective, Theorem 1 states that communication systems in equilibrium cannot pass any information, which was also proven in [12].

If the channel is stable and the information sequence sent by the transmitter is independent and identically distributed, then for every moment and given the state of transmitter, the conditional Shannon entropy H(Y|X) of the receiver is equal, i.e., ΔS=0. Therefore, by combining Equations (1) and (5), the minimal energy dissipation of the communication system is

(6) $$Q_{\min}=kT\cdot I\left(X;Y\right).$$

Equation (6) can be considered a specific version of Landauer’s principle, demonstrating the close relationship between information and energy. It emphasizes the importance of thermodynamics in information transmission. According to Landauer’s principle, erasing one bit of information requires a minimum energy of kTln2. Similarly, Equation (6) indicates that the minimal energy dissipation for transmitting information is directly proportional to the mutual information between the transmitter and receiver. This thermodynamic perspective underscores the fundamental limits of energy efficiency in communication systems.

## 3. Communication Heat Engine

**Figure 1:**
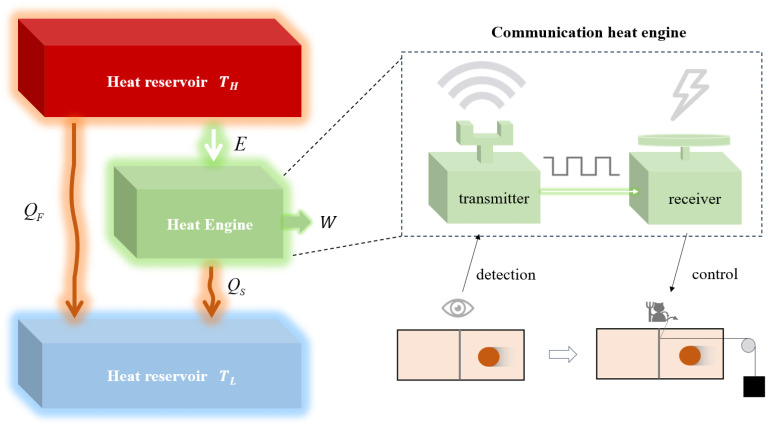
Schematic model of the communication heat engine.

Similar to Maxwell’s demon, which relies on information to extract energy from a single heat reservoir, a communication system can also make use of the information it transmits to obtain a specific amount of energy. For example, by leveraging the information learned about the sun and the environment, communication systems can be integrated with solar thermal power generation systems to help track the position of the sun and concentrate the solar radiation. Therefore, information can be considered a valuable resource for indirectly producing electricity, reducing thermal losses, and enhancing the energy efficiency of power generators [38]. From the perspective of information thermodynamics, each bit of information corresponds to a specific value of energy. Based on this idea, we propose a communication heat engine model to describe a theoretical communication system, for which the communication system is not an information system whose sole purpose is the transmission of information, but a thermodynamic system that utilizes the information and energy in a unified way. The communication heat engine model is shown in Figure 1, where the high-temperature heat reservoir with temperature $T_{H}$ refers to a kind of energy source, such as solar energy, and the low-temperature heat reservoir with temperature $T_{L}$ refers to the environment in which the communication system is located. It is determined that $T_{L}\leq T_{H}$. In each working cycle of the engine, we assume that the communication heat engine absorbs a specific quantity of energy *E* from the high-temperature heat reservoir in an isothermal reversible manner [39]. Hence, the entropy loss of the high-temperature heat reservoir is $E/T_{H}$. Inspired by the principles underlying Maxwell’s demon [23], we can identify the entropy loss of the heat source as an amount of information *I*. This amount of information is measured by the communication system transmitter and must be transmitted to the receiver to instruct the receiver to accept the energy from the high-temperature heat reservoir. Hence, the energy that the communication heat engine can absorb in each working cycle can be expressed as [39]

(7) $$E=kT_{H}I.$$

The work produced by the communication heat engine can be described in a more concrete way with the help of a Szilard engine, as shown in Figure 1. The Szilard engine consists of a single particle prepared in a box and a piston in the middle. The box is in contact with a heat bath, and the particle performs an isothermal quasi-static expansion. Through detection, the transmitter obtains the information about which side the particle is trapped. The receiver receives the information from the transmitter and then uses this information to extract positive work by controlling on which side of the piston the weight is attached. It is worth pointing out that although the communication heat engine model proposed here is a physical realization of Maxwell’s demon, the conversion of information to energy can be achieved in a “demonless” manner as well [40].

As a type of irreversible heat engine, the heat flow to the low-temperature heat reservoir consists of two parts. The first part is the internal energy dissipation of the communication heat engine, which is denoted as $Q_{S}$. The internal energy dissipation includes the heat dissipation of the receiver with a minimal amount of $Q_{\min}=T_{L}I$ and the signal dissipation caused by the deterioration of the signal during transmission in the communication channels. The second part is the external frictional loss of the heat engine, which is define as the energy cost of extra information processing of the communication system and is denoted by $Q_{F}$. According to the law of energy conservation, the work output of the communication heat engine is $W=E-Q_{S}$, and the absolute energy efficiency of the communication heat engine is defined by

(8) $$\eta =\frac{E-Q_{S}}{E+Q_{F}}.$$

If we ignore the deterioration of the signal transmitted in the communication channel and the frictional loss of the communication system, the maximum energy efficiency of the communication heat engine is

(9) $$\eta_{\max}=\frac{E-Q_{\min}}{E}=1-\frac{T_{L}}{T_{H}},$$

which is the Carnot efficiency. Therefore, the energy efficiency of the communication heat engine satisfies 0≤η≤1. It should be noted that energy efficiency is often quantified in terms of bits per joule (bit/J) in communication systems, which measures the number of bits transmitted per unit of energy consumed. This metric provides a direct indication of how efficiently a communication system uses energy to transmit information. In our paper, energy efficiency is derived from a thermodynamic perspective. It is related to the irreversibilities and inefficiencies in the system, indicating how effectively communication systems convert information into useful work. The energy dissipation in the definition of the proposed energy efficiency represents the actual energy lost during the communication process. Reducing energy dissipation directly improves the bit/J efficiency. More importantly, the dimensionless nature of energy efficiency reveals the physical connection between information and energy in communication systems.

Assuming that a time duration of τ is needed for the communication heat engine to complete a working cycle, which is equal to the time required for the transmission of the information, i.e., τ=I/R, where *R* is the information transmission rate measured in nats per second, we can further obtain the output power *P* of the communication heat engine, which is defined as the information power and calculated as

(10) $$P=\lim_{\tau \to \infty }\;\frac{E-Q_{S}}{\tau }.$$

It should be noted that the above limit is the ergodic version of the law of large numbers. Using this definition, despite the random nature of the input energy to the communication heat engine, the information power becomes a deterministic entity under the framework of classical thermodynamics.

## 4. Information Power of the Engine

Unlike the definition of information transmission rate in Shannon’s information theory, here, we use a new method from thermodynamics to derive the information transmission rate of the communication heat engine, thus obtaining the expression of the information power. For a realistic wired or wireless communication system, the transmission of information is achieved through the sending and receiving of signals. We assume the transmitted signal is an electrical signal with the bandwidth *B* and a time interval of τ, and it can be represented mathematically by a function of the voltage with respect to time. In the time domain, the signal can be expanded into an infinite number of mutually orthogonal sub-signals by means of Fourier series. In the frequency domain, the signal has corresponding sine/cosine coefficients at each frequency point, and the interval between each frequency point is 1/τ. Therefore, within the bandwidth of *B* and the time interval of τ, the number of total frequency points of transmission signal is n=2Bτ. The number of signal frequency points means that there are *n* distinct configurations the signal can occupy within the bandwidth *B* and the time interval τ. Therefore, in this article, we define *n* as the degree of freedom of the transmission signal in the communication heat engine. Considering the power of the transmitted signal to be $P_{X}$, when passing through the communication channel, the power of the signal is attenuated. If we assume that the attenuation coefficient of the channel is α, and the power of the received signal by the receiver is $\alpha P_{X}$, then the signal dissipation of the communication heat engine is $\left(1-\alpha \right)P_{X}\tau$. Shannon’s channel coding theorem shows that for any channel, if the information transmission rate is less than the channel capacity of that channel, there exists a coding method that allows the system’s error probability to be arbitrarily small when the code length is sufficiently long [41]. In this article, we assume that the channel of the communication system is an additive-noise channel and that the thermal noise and signal are independent of each other, and that the communication system adopts the coding and decoding method such that the receiver’s error probability tends to zero.

Consider the received signal at the receiver to be a thermodynamic system to be analyzed. Within the time interval τ, the amount of information obtained by the receiver is defined as the entropy change associated with the energy of the received signal $\Delta S_{Y}$. This can be expressed as

(11) $$I\equiv \Delta S_{Y}=\int_{U_{0}}^{U_{1}}\frac{dU}{T_{Y}},$$

where $T_{Y}$ is defined as the signal temperature of the received signal, and *U* is the average energy of the signal system calculated over the time interval τ. The signal temperature is dependent on the average energy of the signal. $U_{0}$ represents the energy of the system when the system is in the initial state of the signal, which means that no information is contained in the transmitted signal and the energy is solely due to thermal noise, i.e., $U_{0}=N\tau$, where *N* is the noise power. $U_{1}$ represents the energy of the system when the system is in the end state of signal transmission, which means that the energy of the system contains the energy of both the received signal and the thermal noise, i.e., $U_{1}=\left(\alpha P_{X}+N\right)\tau$. Moreover, in Equation (11), it is assumed that the energy increase process of the signal system is a step-wise process for the communication heat engine.It should be noted that the definition of information in Equation (11) is based on the premise that the information transmission in the communication heat engine is realized through the signal with energy. In the thermodynamic analysis of information processing, the information can be assumed to be carried in the memory register with no energy [42,43].

**Theorem** **2.** *The average energy of a signal system is given by half the number of its degrees of freedom (in natural units), i.e., $U=nT_{Y}/2$.*

**Proof.**  See Appendix A. □

Based on Theorem 2 and referring to Equation (11), the amount of received information during the time τ is

(12) $$I=\int_{U_{0}}^{U_{1}}\frac{n}{2}\frac{dU}{U}=\frac{n}{2}\log\frac{U_{1}}{U_{0}}=\frac{n}{2}\log\left(1+\frac{\alpha P_{X}}{N}\right)$$

Therefore, within the framework of thermodynamics in Figure 1, the information transmission rate of the communication heat engine is

(13) $$R=\frac{I}{\tau }=\frac{n}{2\tau }\log\left(1+\frac{\alpha P_{X}}{N}\right)=B\log\left(1+\frac{\alpha P_{X}}{N}\right).$$

It is obvious that for a transmitted signal with a fixed bandwidth and transmission power, the information transmission rate is maximal when there is no channel attenuation, i.e., α=1. Therefore, the maximal information transmission rate is $B\log\left(1+P_{X}/N\right)$, which is identical to the Shannon capacity. In the remaining part of our article, we assume that the heat dissipation of the receiver is $T_{L}I$. Therefore, the internal energy dissipation of the communication heat engine is the sum of the receiver heat dissipation and signal dissipation in the time interval τ, which is expressed as

(14) $$Q_{S}=kT_{L}I+\left(1-\alpha \right)P_{X}\tau .$$

Substituting Equations (13) and (14) into Equation (10), the information power of the communication heat engine is obtained:

(15) $$P=kT_{H}R-\frac{Q_{S}}{\tau }=kB\left(T_{H}-T_{L}\right)\log\left(1+\frac{\alpha P_{X}}{N}\right)-\left(1-\alpha \right)P_{X}.$$

Given the significance of both entropy and energy in the communication heat engine, the Boltzmann constant is kept explicit in the expression of information power. This helps to maintain clarity between the kinematical and entropic descriptions of information, as highlighted in [44]. It can be seen from Equation (15) that the information power is decided by the power and bandwidth of the received signal, the temperatures of the heat reservoirs, and the attenuation coefficient of the communication channel. Consequently, within a fixed time interval, transmitting a greater amount of information will result in larger information power produced by the communication heat engine. Moreover, the relationship between the information power and signal power is not monotonic. Because the energy dissipated during the transmission of the signal increases linearly as the signal power increases, whereas the energy gained by the communication heat engine using the information increases only logarithmically, the information power increases first and then decreases with the signal power. The derivative of signal power in Equation (15) is

(16) $$\frac{\partial P}{\partial P_{X}}=\frac{\alpha kB\left(T_{H}-T_{L}\right)}{N+\alpha P_{X}}-\left(1-\alpha \right).$$

Setting Equation (16) as zero, it is easy to determine that the information power of the communication heat engine achieves its maximal value when the signal power is $P_{X}=\frac{kB\left(T_{H}-T_{L}\right)}{1-\alpha }-\frac{N}{\alpha }$. And the maximal information power is expressed as

(17) $$P_{\max}=k\left(T_{H}-T_{L}\right)B\log\frac{B\left(T_{H}-T_{L}\right)}{N(1-\alpha )}-k\left(T_{H}-T_{L}\right)B+\frac{1-\alpha }{\alpha }N.$$

## 5. Multi-Channel Communications

**Figure 2:**
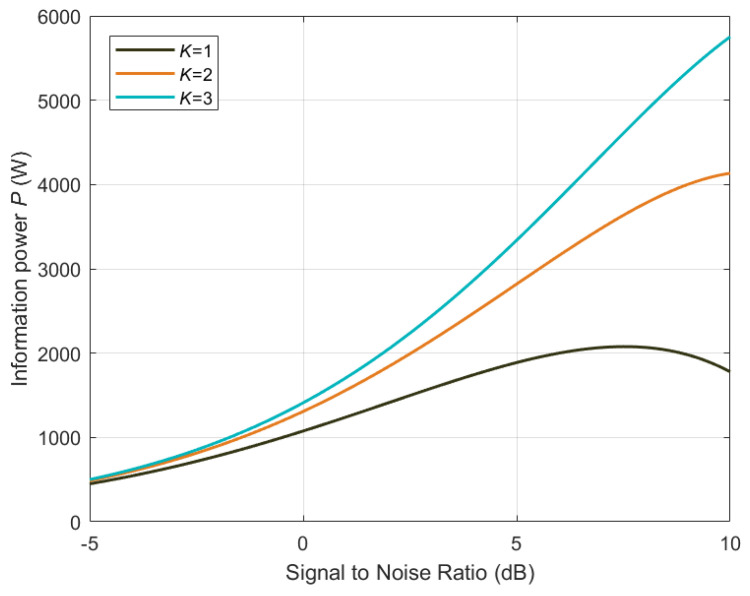
Information power under multi-channel communications.

In the above discussions, we considered the point-to-point communication process of the communication heat engine. In the actual design of the communication heat engine, we can increase the information transmission rate by using multiple channels for communication systems. In this case, the communication heat engine can be considered to be a composite communication system consisting of multiple sub-communication heat engines operating in parallel. The transmission of information inside each sub-engine is achieved by a communication channel. All communication channels in the communication heat engine are assumed to be independent of each other, and the temperature of heat reservoirs in each sub-communication heat engine is assumed to be identical. The parallel transmission mechanism aligns with the concept of the Orthogonal Frequency Division Multiplexing (OFDM) scheme in modern communication systems. OFDM divides the communication channel into multiple orthogonal sub-channels, allowing for parallel data transmission and improving the spectral efficiency. Each sub-channel in OFDM can be viewed as a separate communication channel within the heat engine framework. Assume there are *K* communication channels used by the communication heat engines, each of which has a bandwidth of B=100Hz, and the total amount of information to be transmitted is *I*. The information to be transmitted by the *i*th channel is denoted by $I_{i}$, and the signal power corresponding to it is denoted by $P_{X_{i}}$, with $\sum_{i}I_{i}=I$ and $\sum_{i}P_{X_{i}}=P_{X}$. According to Equation (15), for each sub-communication heat engine, the information power is dependent on the information transmission rate and the signal power transmitted in the communication channel. Therefore, the total information power of the communication heat engine using *K* communication channels can be expressed as

(18) $$P_{\mathrm{total}}=\sum_{i=1}^{K}kR_{i}\left(T_{H}-T_{L}\right)-\left(1-\alpha \right)P_{X},$$

where the information transmission rate in each channel is $R_{i}=B\log\left(1+\alpha S_{i}/N\right)$. When the total signal power is fixed, the information power is maximized by allocating the signal power for each communication channel evenly (See Appendix B). Unless otherwise specified, the signal power allocation in the rest of this article is the optimal case of even allocation. The relationship between the information power and the Signal-to-Noise Ratio (SNR) of the receiver at different channel numbers is shown in Figure 2, where the attenuation coefficient α is set to 0.99. It can be seen from the results in Figure 2 that under the given range of the SNR, the information power of the communication heat engine with a single communication channel is not a monotonical function of the SNR. Given the same value of the SNR, the information power of the communication heat engine with multiple channels is greater than the information power of the communication heat engine with single channel. As the number of channels increases, the information power of the communication heat engine is improved. For all the cases shown in Figure 2, the information power of the communication heat engine eventually goes to zero when the signal power increases to infinity. This is because the signal power allocated to each channel is infinite and the signal dissipation of each sub-communication heat engine is also infinite. According to Equation (15), the communication heat engine can no longer perform any useful work. The results in Figure 2 also imply that optimizing the power allocation in OFDM systems can reduce the energy dissipation, thereby enhancing the system performance and conserving energy.

For realistic communication systems, there is an extra energy cost for multi-channel communications, e.g., the implementation of extra antennas and processing units in Multiple Input–Multiple Output (MIMO) communication systems. Denote the extra energy cost within an unit time as $P_{K}$, which can be regarded as a part of the friction dissipation. The energy efficiency of the communication heat engine considering the friction dissipation is defined as

(19) $$\eta =\frac{E-Q_{S}}{E+Q_{F}}=\frac{kR\left(T_{H}-T_{L}\right)-\left(1-\alpha \right)P_{X}}{kRT_{H}+P_{K}}.$$

**Figure 3:**
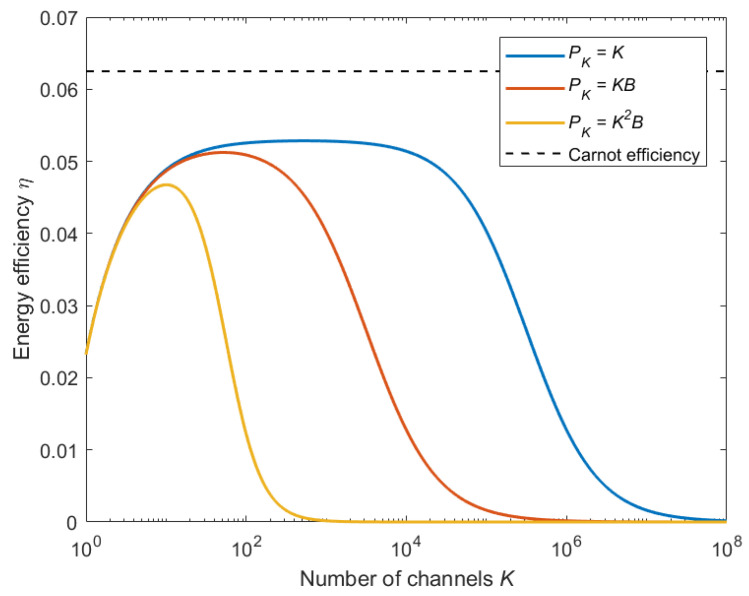
Energy efficiency of multi-channel communication heat engine.

The relationship between the energy efficiency and the number of channels is shown in Figure 3, where different scaling behaviors of $P_{K}$ with respect to *K* are investigated for the following examples: (i) circuit components whose power model [9] is linear in *K* (Figure 3, blue line); (ii) a linear processing scheme with the maximum ratio transmission [45], whose power cost is proportional to KB (Figure 3, red line); (iii) minimum mean square error (MMSE) channel estimation [9], which consumes power proportionally to $K^{2}B$ (Figure 3, yellow line). The friction dissipation model of signal processing is based on the computational complexity and the assumption that each transmitter antenna corresponds to a communication channel. It can be observed that the energy efficiency of the communication heat engine first increases then decreases with the number of channels. In Figure 3, the Carnot efficiency is also drawn. The gap between the absolute energy efficiency of the communication heat engine and the Carnot efficiency is caused by signal dissipation and friction dissipation. Specifically, when the number of channels tends to infinity, the energy efficiency converges to zero. This provides an explicit explanation of why the number of antennas in MIMO communication systems cannot increase without limit. Moreover, from a design perspective, our findings indicate that there is an optimal number of antennas where the energy efficiency is maximized. The design of MIMO communication systems could be enhanced by balancing the trade-off between the number of antennas and energy efficiency.

## 6. Impact of Inter-Channel Interference

When multiple channels are utilized for communication systems, interference between channels must be considered. Usually, the greater the number of channels, the more serious the interference. Inter-channel interference not only affects the communication quality but also reduces the efficiency of the communication heat engine. We denote the interference power of a communication channel as $N_{\mathrm{mc}}$, whose exact value depends on many factors, such as the signal power, number of channels, precoding scheme, etc. [46]. The information transmission rate of the *i*th channel of the communication heat engine becomes

(20) $$R_{i}=B\log\left(1+\frac{\alpha P_{X_{i}}}{N+N_{\mathrm{mc}}}\right).$$

Without loss of generality, the interference factor of the communication heat engine is defined as

(21) $$\gamma =\frac{N_{\mathrm{mc}}}{N+N_{\mathrm{mc}}}.$$

The interference factor is the ratio of the inter-channel interference power to the addition of noise power and the interference power, which satisfies 0≤γ<1. For a communication heat engine with *K* communication channels, the total information power can be written as

(22) $$\begin{array}{cc} P_{\mathrm{total}} & =\sum_{i=1}^{K}kR_{i}\left(T_{H}-T_{L}\right)-\left(1-\alpha \right)P_{X}\\ \; & =\sum_{i=1}^{K}kB\left(T_{H}-T_{L}\right)\log\left(1+\frac{\alpha P_{X_{i}}}{N+N_{\mathrm{mc}}}\right)-\left(1-\alpha \right)P_{X}\\ \; & =kKB\left(T_{H}-T_{L}\right)\log\left(1+\frac{\alpha P_{X}\left(1-\gamma \right)}{NK}\right)-\left(1-\alpha \right)P_{X} \end{array}$$

Based on Equations (20) and (22), an increase in the interference factor will reduce the information transmission rate in the communication channel, hence reducing the total information power of the communication heat engine.

If we set $P_{\mathrm{total}}=0$, the interference factor can be calculated as

(23) $$\gamma =1-\frac{\left(\exp\left(\frac{\left(1-\alpha \right)P_{X}}{KB(T_{H}-T_{L})}\right)-1\right)NK}{\alpha P_{X}}.$$

For computation convenience, we define $m=\frac{\left(1-\alpha \right)P_{X}}{B(T_{H}-T_{L})}$. Therefore, the numerator of Equation (23) can be written as $(e^{m/K\;}-1)K$. By defining κ=1/K and applying L’Hôpital’s rule, we obtain

(24) $$\begin{array}{cc} \lim_{K\to \infty }\left(e^{m/K\;}-1\right)K & =\lim_{\kappa \to 0}\frac{e^{m\kappa }-1}{\kappa }\\ \; & =\lim_{\kappa \to 0}me^{m\kappa }\\ \; & =m. \end{array}$$

Substituting Equation (24) into Equation (23), the interference factor is

(25) $$\gamma^{*}=1-\frac{N(1-\alpha )}{\alpha B(T_{H}-T_{L})},$$

which is defined as the the critical value of the interference factor.

**Figure 4:**
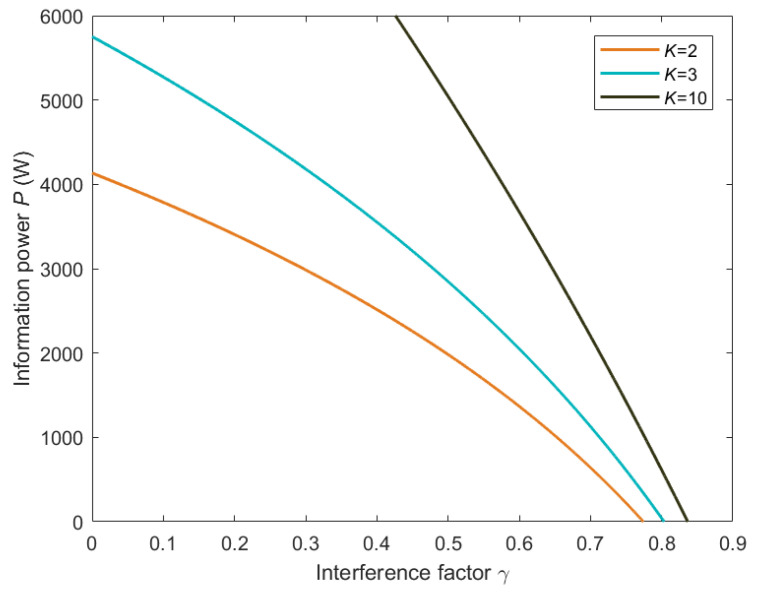
Relationship between information power and interference factor.

The effect of inter-channel interference on the information power of the communication heat engine with different channel numbers is shown in Figure 4, where γ=0 represents the multi-channel communication heat engine without interference. Based on results in Figure 4, the larger the interference factor, the smaller the information power output by the communication heat engine. In particular, when the interference factor is greater than the critical value of the interference factor, the information power of the communication heat engine will become negative, which means that the communication heat engine is no longer able to perform work. It is shown in Figure 4 that under the condition of the same interference factor, the greater the number of channels, the greater the information power of the communication heat engine.

Multi-channel communication heat engines can be considered to be communication systems implementing multiple-access (MA) techniques, where user interference can be measured by the interference factor of the communication heat engine. MA techniques such as Code Division Multiple Access (CDMA) and Non-Orthogonal Multiple Access (NOMA) enable multiple users to share the same communication resources efficiently. The results in Figure 4 highlight the critical role of interference control in MA systems. By reducing user interference, the information power of the communication heat engine can be significantly improved, leading to more efficient and effective communication systems.

## 7. Discussion

Theories of information and communications have traditionally been studied in a mathematical way. Based on tools from stochastic thermodynamics, rigorous physical frameworks and studies on the communication process have started to emerge recently [47,48]. Contrary to the analogy of a communication system with the reversible Carnot engine model [49], the communication heat engine proposed in this article considers irreversible processes occurring within the engine. Entropy production is an important quantity used in our formalism, which measures how irreversible the communication process is. It is worth pointing out that entropy production could be regarded as a conserved quantity just as energy itself is conserved [50]. Furthermore, it has been demonstrated that energy and entropy production are closely related, with one being the (Wick-rotated) complex conjugate of the other in natural units [51]. Although our treatment of entropy production is not complex, mentioning these works provides a supportive context for our findings about the relationship between energy dissipation and entropy production in communication systems.

It should be noted that although there are plenty of studies which consider the joint transmission of information and energy in the wireless communication field [52,53,54,55], the communication heat engine model proposed in this article illustrates the thermodynamic role of information in a communication system. The model provides a theoretical communication mechanism where the information and energy are transmitted not only simultaneously but also in a fully parallel manner. The key point of this model is that the information can be treated as a kind of thermodynamic fuel to perform work. The information power of the communication heat engine is derived based on the concept of the degree of freedom of a signal system. A similar approach is used in the analysis of entropy production associated with beta-decay nuclear disintegration [56].

An approach to improving the information power of the communication heat engine is proposed by implementing multi-channel communications, as shown in Figure 2. Similar ideas can be found in MIMO communication systems [57] and biological communication systems [58], which utilize the diversity of different communication channels to enhance the efficiency and reliability of the system. However, the energy efficiency of the communication heat engine will approach zero when the number of channels tends to infinity. Moreover, this advantage can only be assured when the interference among the channels is not large. The optimal deployment of the communication heat engine needs to be carefully considered with respect to channel conditions. We expect that communication mechanisms based on the communication heat engine can help in the design of future communication system that handle information and energy in a more unified and efficient way.

## 8. Conclusions

In this article, a simplified approach is used to derive the minimal entropy production of a communication process, demonstrating the nonequilibrium nature of communication systems. Based on the result, we model the communication system as a communication heat engine which can perform useful work by utilizing the transmission of information. The output power of the communication heat engine is defined as the information power. Considering the thermodynamics of the Shannon information flow, the information transmission rate and the information power are derived within the framework of classical thermodynamics. From the perspective of the communication heat engine, the dimensionless energy efficiency of communication systems is defined, and the relationships between information power, communication channels, and inter-channel interference are investigated. Our findings constitute a step toward understanding the physical relationships between information and energy in communication systems.

## Data Availability

The datasets used or analyzed during the current study are available from the corresponding author on reasonable request.

## Appendix A

We assume that the transmitted signal is a periodic signal with period τ and a finite bandwidth of *B*. Using the Fourier series, the signal can be represented by a total *n* number of in-phase and orthogonal frequency points of the signal. The interval of the frequency points is 1/τ.

According to Parseval’s theorem [59], the energy of a signal is equal to the sum of the squares of coefficients at all frequency points. Without losing generality, the signal energy per degree of freedom is denoted as $\varpi v^{2}$, where ϖ is a constant coefficient, and *v* is the amplitude of each frequency point. The energy of signal *u* is the sum of the signal energy per degree of freedom, i.e., $u=\sum_{j=1}^{n}\varpi_{j}v_{j}^{2}$. The probability distribution of the signal energy is assumed to satisfy the Boltzmann distribution, which is

(A1) $$p_{u}=\frac{\exp\left(-\beta_{Y}\sum_{j=1}^{n}\varpi_{j}v_{j}^{2}\right)}{\int \cdots \int \exp\left(-\beta_{Y}\sum_{j=1}^{n}\varpi_{j}v_{j}^{2}\right)dv_{i}\cdots dv_{n}},$$

where $\beta_{Y}=1/kT_{Y}$, *k* is the Boltzmann constant and $T_{Y}$ is the signal temperature.

It should be noted that the transmitted signal is generally under nonequilibrium conditions in real-world communication scenarios. The Boltzmann distribution of signal energy is adopted as a simplifying assumption to maximize the information transmission rate based on the information definition in this article. Different from the analysis of the energy dissipation of the communication system, where the conditional energy of the receiver appears in the Boltzmann distribution, here, the transmitted signal itself is considered an independent thermodynamic system. Consequently, the Boltzmann distribution of signal energy is a non-conditional one. Based on Equation (A2), the average energy of the signal is

(A2) $$\begin{array}{cc} U & =\int \cdots \int up_{u}dv_{1}\cdots dv_{n}\\ \; & =\frac{\int \cdots \int \left(\sum_{i=1}^{n}\varpi_{i}v_{i}^{2}\right)\exp\left(-\beta_{Y}\sum_{j=1}^{n}\varpi_{j}v_{j}^{2}\right)dv_{i}\cdots dv_{n}}{\int \cdots \int \exp\left(-\beta_{Y}\sum_{j=1}^{n}\varpi_{j}x_{j}^{2}\right)dv_{i}\cdots dv_{n}}\\ \; & =\sum_{i=1}^{n}\frac{\int \cdots \int \varpi_{i}v_{i}^{2}\exp\left(-\beta_{Y}\sum_{j=1}^{n}\varpi_{j}v_{j}^{2}\right)dv_{i}\cdots dv_{n}}{\int \cdots \int \exp\left(-\beta_{Y}\sum_{j=1}^{n}\varpi_{j}v_{j}^{2}\right)dv_{i}\cdots dv_{n}}\\ \; & =\sum_{i=1}^{n}\frac{\int \varpi_{i}v_{i}^{2}\exp\left(-\beta_{Y}\varpi_{i}v_{i}^{2}\right)dv_{i}}{\int \exp\left(-\beta_{Y}\varpi_{i}v_{i}^{2}\right)dv_{i}}\\ \; & =\sum_{i=1}^{n}\frac{1}{2\beta_{Y}}=\frac{1}{2}nkT_{Y}, \end{array}$$

where *n* is the degree of freedom of the received signal. Equation (A3) shows that the average energy of a time domain signal is only related to its degree of freedom and temperature.

## Appendix B

Considering the number of communication channels to be *K*, the bandwidth of each channel is fixed as *B*, and the total signal power is *S*. The information power of the communication heat engine is

(A3) $$P_{\mathrm{total}}=\sum_{i=1}^{K}\left(R_{i}\left(T_{H}-T_{L}\right)-\left(1-\alpha \right)P_{X_{i}}\right),$$

where $R_{i}=B\log\left(1+\frac{\alpha P_{X_{i}}}{N}\right)$, $P_{X_{i}}$ and $R_{i}$ are the signal power and information transmission rate of the *i*th channel, respectively. Based on Equation (A3), the total information power of a communication heat engine with two communication channels is calculated as

(A4) $$\begin{array}{cc} P_{\mathrm{total}} & =kR_{1}\left(T_{H}-T_{L}\right)-\left(1-\alpha \right)P_{X_{1}}+kR_{2}\left(T_{H}-T_{L}\right)-\left(1-\alpha \right)P_{X_{2}}\\ \; & =k\left(R_{1}+R_{2}\right)\left(T_{H}-T_{L}\right)-\left(1-\alpha \right)P_{X}\\ \; & =kB\log\left(\left(1+\frac{\alpha P_{X_{1}}}{N}\right)\left(1+\frac{\alpha P_{X_{2}}}{N}\right)\right)\left(T_{H}-T_{L}\right)-\left(1-\alpha \right)P_{X}, \end{array}$$

where we have

(A5) $$\left(1+\frac{\alpha P_{X_{1}}}{N}\right)\left(1+\frac{\alpha P_{X_{2}}}{N}\right)=\frac{1}{4}\left({\left(2+\frac{\alpha P_{X}}{N}\right)}^{2}-{\left(\frac{\alpha \left(P_{X_{1}}-P_{X_{2}}\right)}{N}\right)}^{2}\right).$$

It can be seen from Equation (A5) that the smaller the difference between the signal power of each channel, i.e., $P_{X_{1}}-P_{X_{2}}$, the greater the total information power. In particular, when the signal power of two communication channels is equal, i.e., $P_{X_{1}}=P_{X_{2}}=\frac{1}{2}P_{X}$, the total information work is maximal. Alternatively, we can rewrite Equation (A5) in terms of an inequality based on the square factorization, which is

(A6) $$\left(1+\frac{\alpha P_{X_{1}}}{N}\right)\left(1+\frac{\alpha P_{X_{2}}}{N}\right)\leq {\frac{\left(\left(1+\frac{\alpha P_{X_{1}}}{N}\right)+\left(1+\frac{\alpha P_{X_{2}}}{N}\right)\right)}{4}}^{2},$$

and the equality holds only when $P_{X_{1}}=P_{X_{2}}=\frac{1}{2}P_{X}$. When the number of channels is *K*, assuming the inequality

(A7) $$\left(1+\frac{\alpha P_{X_{1}}}{N}\right)\cdots \left(1+\frac{\alpha P_{X_{K}}}{N}\right)\leq {\frac{\left(\left(1+\frac{\alpha P_{X_{1}}}{N}\right)+\left(1+\frac{\alpha P_{X_{2}}}{N}\right)+\cdots +\left(1+\frac{\alpha P_{X_{K}}}{N}\right)\right)}{K^{K}}}^{K},$$

holds, the equality holds only when $P_{X_{1}}=P_{X_{2}}=\cdots =P_{X_{K}}=\frac{1}{K}P_{X}$. When the number of channels becomes K+1, by setting $S_{K+1}$ as the maximal signal power among all the signal powers allocated in K+1 communication channels and recalling the fact that $P_{X_{1}}+P_{X_{2}}+\cdots +P_{X_{K}}+P_{X_{K+1}}=P_{X}$, it follows that

(A8) $$\begin{array}{cc} \; & K\left(1+\frac{\alpha P_{X_{K+1}}}{N}\right)\geq \left(1+\frac{\alpha P_{X_{1}}}{N}\right)+\left(1+\frac{\alpha P_{X_{2}}}{N}\right)+\cdots +\left(1+\frac{\alpha P_{X_{K}}}{N}\right)\\ \; & =K+\frac{\alpha \left(P_{X}-P_{X_{K+1}}\right)}{N}. \end{array}$$

Based on Equations (A7) and (A8), we can write

(A9) $$\begin{array}{cc} \; & \left(1+\frac{\alpha P_{X_{1}}}{N}\right)\left(1+\frac{\alpha P_{X_{2}}}{N}\right)\cdots \left(1+\frac{\alpha P_{X_{K+1}}}{N}\right)\\ \; & \overset{\left(a\right)}{\leq }\;{\left(\frac{K+\frac{\alpha \left(P_{X}-P_{X_{K+1}}\right)}{N}}{K}\right)}^{K}\left(1+\frac{\alpha P_{X_{K+1}}}{N}\right)\\ \; & ={\left(\frac{K+\frac{\alpha \left(P_{X}-P_{X_{K+1}}\right)}{N}}{K}\right)}^{K+1}\\ \; & +\left(K+1\right){\left(\frac{K+\frac{\alpha \left(P_{X}-P_{X_{K+1}}\right)}{N}}{K}\right)}^{K}\frac{K\left(1+\frac{\alpha P_{X_{K+1}}}{N}\right)-\left(K+\frac{\alpha \left(P_{X}-P_{X_{K+1}}\right)}{N}\right)}{K\left(K+1\right)}\\ \; & \overset{\left(b\right)}{\leq }\;{\left(\frac{K+\frac{\alpha \left(P_{X}-P_{X_{K+1}}\right)}{N}}{K}+\frac{K\left(1+\frac{\alpha P_{X_{K+1}}}{N}\right)-\left(K+\frac{\alpha \left(P_{X}-P_{X_{K+1}}\right)}{N}\right)}{K\left(K+1\right)}\right)}^{K+1}\\ \; & ={\left(\frac{\left(1+\frac{\alpha P_{X_{1}}}{N}\right)+\left(1+\frac{\alpha P_{X_{2}}}{N}\right)+\cdots +\left(1+\frac{\alpha P_{X_{K+1}}}{N}\right)}{K+1}\right)}^{K+1}, \end{array}$$

where (a) holds because of the assumption of Equation (A7), and (b) holds because of the binomial expansion. The equality in (b) holds only when equation $K\left(1+\frac{\alpha P_{X_{K+1}}}{N}\right)=K+\frac{\alpha \left(P_{X}-P_{X_{K+1}}\right)}{N}$ is satisfied. Therefore, only when $P_{X_{1}}=P_{X_{2}}=\cdots =P_{X_{K+1}}=\frac{1}{K+1}P_{X}$ is satisfied do the equalities in Equation (A9) hold. This proves that only when the signal power of each communication channel is evenly allocated is the information power of the communication heat engine maximal.
